# EX-MET study: exercise in prevention on of metabolic syndrome – a randomized multicenter trial: rational and design

**DOI:** 10.1186/s12889-018-5343-7

**Published:** 2018-04-02

**Authors:** Arnt Erik Tjønna, Joyce S. Ramos, Axel Pressler, Martin Halle, Klaus Jungbluth, Erika Ermacora, Øyvind Salvesen, Jhennyfer Rodrigues, Carlos Roberto Bueno, Peter Scott Munk, Jeff Coombes, Ulrik Wisløff

**Affiliations:** 1 K.G. Jebsen Center of Exercise in Medicine at Department of Circulation and Medical Imaging, Faculty of Medicine, Norwegian University of Science and Technology, Trondheim, Norway; 2 School of Human Movement and Nutrition Sciences, University of Queensland, Brisbane, Queensland Australia; 3 Department of Prevention, Rehabilitation and Sports Medicine, Klinikum rechts der Isar, Technische Universität München, Munich, Germany; 4 KJ Fisiosport, Guayaquil, Ecuador; 5 Department of Public Health and General Practice, Norwegian University of Science and Technology, Trondheim, Norway; 6 School of Physical Education and Sport of Ribeirao Preto, University of São Paulo, Ribeiro Preto, Brazil; 7 Sørlandet Hospital HF, Kristiansand, Norway; 8 DZHK (German Center for Cardiovascular Research), partner site Munich Heart Alliance, Munich, Germany; 9 Clinic of Cardiology, St. Olavs Hospital, Trondheim, Norway; 10 Department of Circulation and Medical Imaging, Faculty of Medicine, Norwegian University of Science and Technology, Prinsesse Kristinas gt.3, 7006 Trondheim, Norway

**Keywords:** Exercise training, Metabolic syndrome, Cardiovascular disease

## Abstract

**Background:**

Metabolic syndrome substantially increases risk of cardiovascular events. It is therefore imperative to develop or optimize ways to prevent or attenuate this condition. Exercise training has been long recognized as a corner-stone therapy for reducing individual cardiovascular risk factors constituting the metabolic syndrome. However, the optimal exercise dose and its feasibility in a real world setting has yet to be established.

The primary objective of this randomized trial is to investigate the effects of different volumes of aerobic interval training (AIT) compared to the current exercise guideline of moderate-intensity continuous training (MICT) on the composite number of cardiovascular disease risk factors constituting the metabolic syndrome after a 16 week, 1-year, and 3-year follow-up.

**Methods:**

This is a randomized international multi-center trial including men and women aged ≥30 years diagnosed with the metabolic syndrome according to the International Diabetes Federation criteria. Recruitment began in August 2012 and concluded in December 2016. This trial consists of supervised and unsupervised phases to evaluate the efficacy and feasibility of different exercise doses on the metabolic syndrome in a real world setting. This study aims to include and randomize 465 participants to 3 years of one of the following training groups: i) 3 times/week of 4 × 4 min AIT at 85–95% peak heart rate (HRpeak); ii) 3 times/week of 1 × 4 min AIT at 85–95% HRpeak; or iii) 5–7 times/week of ≥30 min MICT at 60–70% HRpeak. Clinical examinations, physical tests and questionnaires are administered to all participants during all testing time points (baseline, 16 weeks and after 1-, and 3-years).

**Discussion:**

This multi-center international trial indeed aims to ease the burden in healthcare/economic cost arising from treating end-stage CVD related conditions such as stroke and myocardial infarction, that could eventually emerge from the metabolic syndrome condition.

**Trial registration:**

Clinical registration number: NCT01676870, ClinicalTrials.gov (August 31, 2012).

## Background

Several lines of evidence suggest that endurance training improves cardiovascular function [1, 2]. Improvements in maximal work capacity are accompanied by several central and peripheral cardiovascular adaptations that are regarded as the signature of the trained state. These adaptive changes include increased maximal oxygen uptake, improved cardiac- and endothelial function, improved lipid profile and increased abundance of capillaries, mitochondria and metabolic enzymes.

Several of these factors are related to the components of metabolic syndrome, including central obesity, hypertriglyceridemia, low HDL cholesterol, hypertension and raised fasting plasma glucose [3]. Currently, about 25% of the US adult population is classified as having the metabolic syndrome, and the prevalence is expected to increase in parallel to the pandemic of obesity and overweight, which affects about 312 million and 1.1 billion people worldwide, respectively [4, 5]. European numbers seems to be similar to that observed in the US. The European Group for the Study of Insulin Resistance (EGIR) reported that among non-diabetic individuals, the frequency of the metabolic syndrome varied from 7 to 36% for men and between 5 to 22% in women aged 40 to 55 years [6]. It is well established that the risk for premature cardiovascular death is increased in individuals with the metabolic syndrome [7, 8]. Data from Katzmarzyk et al. [2] demonstrate a relative risk of 1.89 (95% CI: 1.36–2.60) for premature cardiovascular mortality in men with metabolic syndrome compared to healthy men. In line with this, combining several factors associated with cardiovascular disease (CVD) gives a more powerful predictor of premature CVD death than assessing separate effects of each factor [4]. Exercise training may reverse most of the risk factors of the syndrome and also protects against premature cardiovascular death [1, 2, 9]. However, the recommendation of moderate physical activity does not seem to prevent the syndrome of reaching pandemic levels and the optimal training regimen remains to be defined.

Today we have good indications that exercise training is an effective contributor for reducing risk factors related to metabolic syndrome [1, 2], but no sufficiently powered, randomized controlled study have documented the contribution of exercise training in the reversal of metabolic syndrome.

## Aims

Primary aim of EX-MET

- The primary aim of the study is to compare the efficacy of traditional training (today’s guideline according to the American College of Sports Medicine [ACSM], vigorously or moderate exercise) and different volumes of aerobic interval training (1-AIT vs. 4-AIT) in reducing risk factors constituting the metabolic syndrome in a real world setting.

Secondary aims of EX-MET

- The secondary objectives are to compare the efficacy of traditional moderate training (ACSM guideline) and different volumes of aerobic interval training (1-AIT vs. 4-AIT) in improving aerobic capacity, cardiovascular function, skeletal muscle contractile function, skeletal muscle energy metabolism, left ventricle systolic and diastolic function at rest and right ventricular function.

## Methods

### Design

**Fig. 1:**
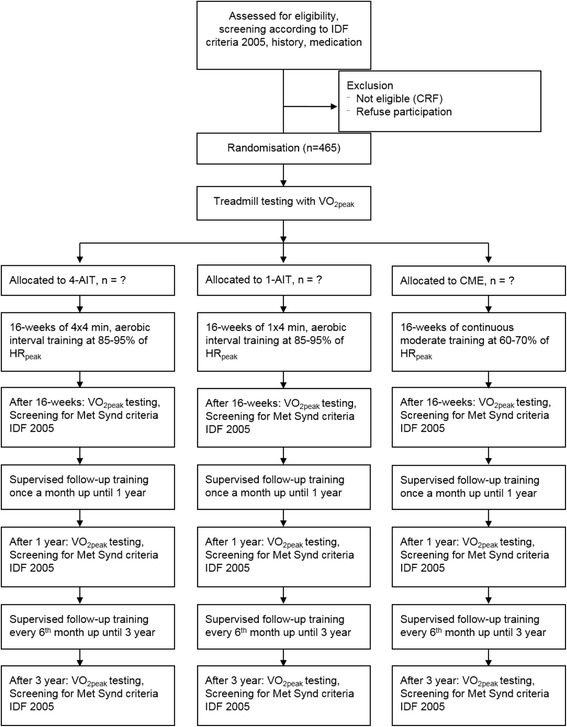
Study design

Participants with the metabolic syndrome defined according to the IDF-criteria [3] are included in this trial. Patients (male and female age ≥ 30) are randomized and stratified (age, gender and center) into the 4x4min aerobic interval training (4-AIT, vigorously exercise according to today’s guidelines [ACSM], three times a week), 1x4min aerobic interval training (1-AIT, three times a week) and moderate-intensity continuous training (MICT, according to ACSM guidelines) groups at a 1:1:1 ratio. This trial consists of two phases. The first phase requires the participants to attend two supervised training sessions with an exercise physiologist per week for 16 weeks, with the residual sessions performed in an unsupervised environment. Participants then enter the second phase of the trial and are instructed to proceed with the same exercise program from the previous 16-week intervention, but in an unsupervised manner. During this phase, they are required to attend a supervised exercise session each month, until the one-year follow-up, and every six months until the 3-year end-point. The participants are tested at baseline, after 16 weeks, and at one-, and three-year follow-up (Fig. 1). The Unit for Applied Clinical Research at the Norwegian University of Science and Technology developed the randomization procedure to ensure impartial assignments. After randomization, participants receive verbal and written information about their intervention. EX-MET has been approved by the Regional Committee for Medical Research Ethics (REK 2011/2150), and was registered in the clinical trials registry in August 2012 (ClinicalTrials.gov, Identifier: NCT01676870). Oral and written informed consent is obtained from all participants before inclusion. Participation in the main study is not influenced by willingness to participate in sub-studies. All sub-studies must have approval from the Regional Committee for Medical Research Ethics and studies will not involve interventions that are in conflict with the main study. Exclusion criteria are unstable angina, recent myocardial infarction (within the last four weeks), uncompensated heart failure, severe valvular illness, pulmonary disease, uncontrolled hypertension, kidney failure, orthopedic/neurological limitations, cardiomyopathy, and planned operations during the research period, reluctant to sign the consent form, drug or alcohol abuse or participants in a parallel study.

### Project organization

The Norwegian University of Science and Technology is the coordinating center of this multi-center trial. This center coordinates and maintains the randomization database, clinical report forms, and adverse effect registry. A steering committee has been appointed to dictate resolutions for major scientific and ethical questions. This committee developed the study protocol and is responsible for data collection, management, and publications derived from this study. Inclusion of participants started in August 2012 and is estimated to finish by the end of 2016. Each center is expected to include at least 50 patients, while the larger sites ≥100.

#### Participating centers

Six centers with local coordinators will include patients:

1. Trondheim (Norway) – Arnt Erik Tjønna
2. Brisbane (Australia) – Jeff Coombes
3. Stavanger (Norway) – Peter Scott Munk
4. Guayaquil (Equador) – Klaus Jungbluth
5. Munich (Germany) – Martin Halle
6. Sao Paulo (Brazil) – Carlos Roberto Bueno Jr.

### Ethics considerations

The risk of exercise is considered small. However, the risk of complications/death is higher during and immediately following training/testing [10]. Nevertheless, the health benefits from exercise training are sufficiently high and are considered less harmful than inactivity. Providing sufficient clear information is a high priority, so that participants feel that they know what consenting entails.

### Examinations

The following examinations are performed at time points indicated above and are described in more detail in our standard operating procedures (Appendix 1). Briefly, blood samples are obtained from an arm vein after a 12-h overnight fast. Triglycerides (TG), glucose, insulin, high-density lipoprotein-cholesterol (HDL-C), low-density lipoprotein-cholesterol (LDL-C), total cholesterol, C-reactive protein (CRP), glycosylated hemoglobin (HbA_1c_), and C-peptide are measured using standard procedures at respective hospitals or centers with high standard of accuracy. Serum and EDTA plasma are centrifuged at 2500 rpm for 10 min at 20 °C. Aliquots are stored at −80°C in case new blood markers will be analyzed later. In addition, whole blood from an EDTA tube is taken and stored (at Regional Biobank) at −80°C for later analysis of DNA and other blood markers. Blood pressure is measured after a 12-h overnight fast and following 15 min of seated quiet rest. Testing of cardiorespiratory fitness (peak oxygen uptake, V̇O_2peak_) is performed on a treadmill or cycle ergometers identical to that in previous studies in our group [11]. Ergometer selected for testing is dependent upon preferred type of exercise during the intervention, as well as physical limitations presented by participants. Participants with previous heart diseases are tested under electrocardiograph (ECG) monitoring, and the ACC/AHA-guidelines for exercise testing of patients with known cardiovascular disease is followed [12]. Body composition and weight are measured using bioelectrical impedance (Inbody 720, BIOSPACE, Seoul, Korea). Physical activity is measured via SenseWear Armband activity monitor (BodyMedia 7, Pittsburgh, PA, USA) or by Actigraph (GT3X, Manufactering Technology Inc., Florida, USA). After the clinical test at the hospital, all participants are given either SenseWear or Actigraph monitor. The participants are instructed to wear it for seven days continuously (24 h), except when in contact with water (shower/bath/swim). Novel genetic biomarkers (DNA and RNA) will be analyzed using high-throughput OMICS technology (genotyping or exome sequencing, DNA methylation, RNA sequencing, microRNA screening, MS-based proteomics, and MR metabolomics) using validated methods established at the Norwegian University of Science and Technology’s Genomics, Proteomics and Metabolomics Core Facilities. Health economic analysis will be calculated as the sum of hospital cost (inpatient, day care, and outpatient care), use of general physician, and primary health care (rehabilitation, nursing homes, home care etc.). Five different questionnaires are used in this study: Questionnaire 1 consisted of questions about specific aspects of physical activity history. The short form health survey SF-8 (1-wk recall version) is used to describe quality of life and chronic pain (Q3). The questions from Q1, Q2 and Q3 are the same questions that have been used in the Nord-Trøndelag Health Study (HUNT) [13–15]. Medications were monitored throughout the study.

### Sample size and statistics

According to estimates based on data from previous studies, [16, 17] a total of 465 patients randomized 1:1:1 to the three interventions gives 80% power to detect an effect of 4-AIT compared to 1-AIT/CME of 51% recovery versus 37% recovery from metabolic syndrome at the 5% significance level. These calculations were based on the following assumptions: The primary endpoint is reduction in the composite number of cardiovascular risk factors constituting the metabolic syndrome (IDF) [3], as a change from baseline to one year follow-up.

### Training interventions

#### Aerobic interval training

**Fig. 2:**
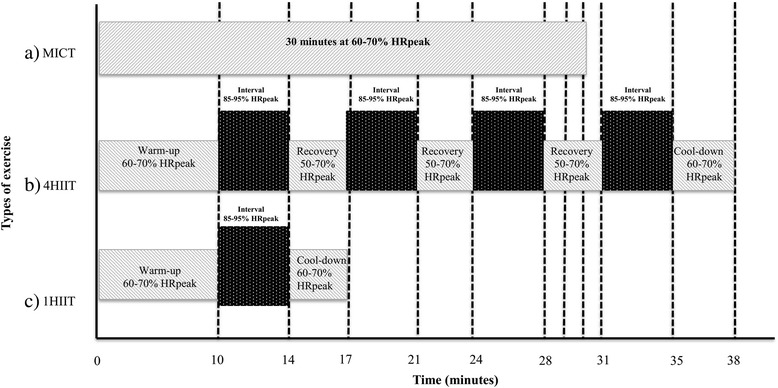
Schematic representation of moderate-intensity continuous training (MICT, **a**) high volume 4 × 4 minutes of high intensity interval training (4-AIT, **b**) and 1 × 4 minute HIIT (1-AIT, **c**)

Participants that are randomized into the aerobic interval training groups (4-AIT and 1-AIT) perform a total of three training sessions per week, for 16 weeks, during the first phase of the trial. Two training sessions are performed in the hosting research institution with an exercise physiologist, and one training session in an unsupervised environment. During the second phase of the trial, all three sessions per week are performed in their own time at home, outdoor or in a gym. Participants are provided with a training log to record all unsupervised training sessions. All training sessions involve activities using large muscle mass. Supervised training sessions are generally performed on a cycle or treadmill ergometer (self-selected). Unsupervised sessions consist of outdoor/indoor pursuits involving different activities such as running, walking, cycling, rowing, swimming, and skiing, performed in the prescribed AIT format. The 4-AIT and 1-AIT groups exercise for 38 and 17 min per session, respectively. Both the AIT protocols include a 10-min warm-up and a 3-min cool-down at 60–70% peak heart rate (HRpeak) or at a rate of perceived exertion [RPE] of 11–13 on a Borg scale. Following the 10-min warm-up, a 4-min interval is performed at 85–95% HRpeak (RPE at 15–17 on Borg scale). This is repeated 4 times in the 4-AIT group, separated by 3 min of active recovery at 50–70% HRpeak (Fig. 2a). The 1-AIT group only performs one 4-min interval at the same intensity and immediately proceeds to the cool-down period (Fig. 2b). The target heart rate should be reached within two minutes of the 4-min interval period for both volumes of AIT (1-AIT and 4-AIT). Heart rate is monitored (Polar electro, Kempele, Finland) during all supervised exercise sessions to ensure participants are reaching the prescribed target heart rate. Participants are encouraged to purchase their own heart rate monitor to keep track of their own training during the unsupervised sessions. Otherwise, they are instructed to use the Borg 6–20 scale (rating of perceived exertion) to serve as a guide towards the exercise intensity achieved [18]. HR and RPE during all training (unsupervised and supervised) sessions are monitored and recorded.

#### Moderate-intensity continuous training

Participants randomized into the MICT group perform 5–7 training sessions per week to conform to the current exercise guideline put forth by the American College of Sports Medicine (ACSM). Two sessions are supervised in an exercise laboratory by an exercise physiologist, and the rest are completed in an unsupervised environment. Training modes for both the supervised and unsupervised sessions are the same as AIT. The MICT group trains continuously for ≥30 min at a target intensity of 60–70% HRpeak (RPE of 11–13 on Borg scale) with no signs of shortness of breath (Fig. 2c). RPE and HR are monitored and recorded for all training sessions as indicated above.

## Discussion

To our knowledge, EX-MET is the first randomized trial to investigate the impact of different volumes of AIT (1-AIT and 4-AIT) and MICT on the treatment of metabolic syndrome in individuals from different ethnic groups. This trial aims to extend previous small studies (*n* = 27 to 40) without a follow-up period that have examined the efficacy of high volume-AIT compared to an iso-caloric MICT on traditional and novel cardiovascular disease (CVD) risk factors [11, 16, 19, 20]. Recent meta-analyses suggest that the greater tendency of high volume-AIT (4-AIT) relative to the traditional MICT to reduce risk of CVD and mortality could be attributable to its greater ability to improve cardiorespiratory fitness [21] and vascular function [22] in clinical populations. Specifically, we have demonstrated a greater impact of 4-AIT compared to an isocaloric MICT in attenuating individual components of the metabolic syndrome, as well as improving cardiorespiratory fitness, vascular function, and mitochondrial function in a small pilot study incorporating 32 individuals diagnosed with metabolic syndrome [16]. We also recently demonstrated that low volume- AIT (1-AIT) is equally as effective as 4-AIT in improving cardiorespiratory fitness in short term in overweight individuals [23]. Thus since cardiorespiratory fitness has been shown to be an antidote towards different CVD risk factors associated with the metabolic syndrome [24], and that ‘lack of time’ has been the most reported barrier to exercise [25], it was logical for us to investigate the impact of low-volume AIT (1-AIT) compared to high-volume dosages of high- (4-AIT) and moderate-intensity exercise (MICT) prescribed according to the current exercise guideline (ACSM). This could help decipher an optimal or alternative, but effective, exercise prescription that we suspect most individuals with metabolic syndrome would better adhere or comply with, in attempt to ease the economic burden of this pandemic.

It is noteworthy that most studies that have investigated the impact of AIT compared to MICT on several factors affecting health in a diverse clinical population have only been from a supervised laboratory setting, conducted over a short-term period (12–16 weeks) [21, 22, 26]. Thus, although most studies revealed a greater tendency for AIT to improve CVD risk factors associated with the metabolic syndrome relative to the current exercise guideline, it is still of question on whether individuals would be able to perform this type of exercise in an unsupervised environment for a long-term period. This multi-center international trial also therefore aims to investigate different strategies that could be implemented in order to increase adherence and compliance to these prescribed exercise interventions in a long-term unsupervised environment. Although having 1–3 sessions performed unsupervised out of the 3–7 sessions required during the 16-week supervised phase of this trial could be considered a limitation of the study, it could also be identified as a strategy to promote adherence and compliance in a ‘real-world setting’. Having specific guidance on what activities are available to implement the prescribed format of exercise (1-AIT, 4-AIT, and MICT) in their own everyday environment, and how to monitor the intensity of training on a weekly basis for the initial 16-weeks could play a huge factor on their motivation and ability to perform the exercise program accurately and safely during the unsupervised phase. Another strategy being enforced in this trial is the requirement of participants to attend a supervised training session each month or every 6-months until the 1-year and 3-year end points, respectively, during the unsupervised phase. If these strategies proves to be effective in increasing both adherence and compliance of participants to the target intervention, then governing bodies around the globe should consider investing on providing vulnerable individuals with exercise-related services incorporated in this trial, such as the initial 16-week consultation with an exercise professional, followed-up with either a monthly/6-monthly visit with an exercise professional, or the use of physical activity and heart rate monitoring devices. This multi-center international trial indeed aims to ease the burden in healthcare/economic cost arising from treating end-stage CVD related conditions such as stroke and myocardial infarction, that could eventually emerge from the metabolic syndrome condition [27].

## Abbreviations

ACSM: American college of sports medicine
AIT: Aerobic interval training
CRP: C-reactive protein
CVD: Cardiovascular disease
EX-MET: Exercise in prevention of metabolic syndrome
HbA_1C_: Glycosylated hemoglobin
HDL: High-density lipoprotein
HR_peak_: Peak heart rate
IDF: International diabetes federation
MICT: Moderate-intensity continuous training
RPE: Rate of perceived exertion
TG: Triglycerides
